# Stimulation of bovine monocyte-derived macrophages with lipopolysaccharide, interferon-ɣ, Interleukin-4 or Interleukin-13 does not induce detectable changes in nitric oxide or arginase activity

**DOI:** 10.1186/s12917-019-1785-0

**Published:** 2019-01-31

**Authors:** Heather Imrie, Diana J. L. Williams

**Affiliations:** 1 Institute of Infection and Global Health, University of Liverpool, 146 Brownlow Hill, Liverpool, L3 5RF UK; 2 Animal, Rural and Environmental Sciences, Nottingham Trent University, Brackenhurst Lane, Southwell, NG25 0DT UK

**Keywords:** Cattle, Macrophage, Nitric oxide, Arginase, Inducible nitric oxide synthase

## Abstract

**Background:**

Bacterial lipopolysaccharide and interferon-γ stimulation of rodent macrophages in vitro induces up-regulation of inducible nitric oxide synthase, whereas interleukin-4 stimulation results in increased activity of arginase-1. Thus different stimulants result in differing macrophage phenotypes, appropriate for responses to a range of pathogens. The current study was conducted in order to determine whether bovine macrophages derived from monocytes and spleen respond similarly.

**Results:**

Lipopolysaccharide and interferon-γ did not induce detectable increases in nitric oxide production by bovine monocyte-derived or splenic macrophages in vitro. Similarly, interleukin-4 and interleukin-13 did not affect arginase activity. However, changes in transcription of genes coding for these products were detected.

**Conclusion:**

Differences between macrophage activation patterns exist between cattle and other species and these differences may occur during the post-transcription phase.

## Background

Macrophages are important effector cells of the immune system, responding to a variety of stimuli by developing diverse phenotypes, capable of differing activities. Mouse and human models suggest that the M1, or classically activated, phenotype is acquired following stimulation by interferon-γ (IFNγ) and/or pathogen-associated molecular patterns (PAMPs) such as lipopolysaccharide (LPS) (reviewed [1]). The M1 phenotype is characterised by the production of reactive oxygen and nitrogen intermediates such as nitric oxide (NO), as well as a range of pro-inflammatory cytokines [2, 3]. This phenotype has a role in immunity to intracellular bacteria with production of NO resulting in damage to bacterial lipids, enzymes and other structures (reviewed [4]). The M2, or alternatively activated, phenotype is acquired following a variety of stimuli, including IL-4 with or without IL-13, the anti-inflammatory cytokines IL-10 and/or TGFβ, immune complexes and uptake of apoptotic cells [5, 6]. M2 macrophages are characterised by the production of L-ornithine (products from which enhance collagen synthesis and healing [7]), chitinase which can digest glycans such as those coating helminths [8] as well as anti-inflammatory/regulatory cytokines and growth factors [9]. The M2 phenotype has a role in controlling helminth health and motility as well as regulating inflammation and stimulating healing [10, 11].

Within macrophages, NO and L-ornithine are both synthesized from the same precursor, L-arginine; the former by the enzyme inducible nitric oxide synthase (iNOS), encoded by the gene nitric oxide synthase 2 (*nos2*) and the latter by arginase-1 encoded by *arg-1* (reviewed [7]). It has been suggested that competition for L-arginine between the two pathways is an important factor in driving cells further along the development of either the M1 or M2 phenotype (reviewed [12]).

In vitro models utilizing rodent macrophages have demonstrated increased production of NO following treatment with the M1 stimulants LPS and IFNγ [7] and increased production of urea (an end product of arginase activity) has been found following stimulation with the M2 stimulant IL-4. These findings are consistent for both monocyte-derived macrophages (MDM) (reviewed [7, 13]) and tissue-resident macrophages such as those from the spleen [14, 15]. However, in vitro models using human MDM or tissue macrophages treated with M1 or M2 stimulants are much less consistent, with some authors failing to note any increase in iNOS or arginase activity following stimulation (reviewed [13, 16]). This lack of consistency in responsiveness may reflect variation in genetics, cell type or previous immunological history and highlights the importance of investigating macrophage activation on an individual species basis. In the current study, we investigated the effects of M1 and M2 stimulants on bovine MDM and splenic macrophages (SM).

## Results

### Effect of stimulation of MDM and SM with cytokines or LPS on M1 and M2 polarisation

**Fig. 1:**
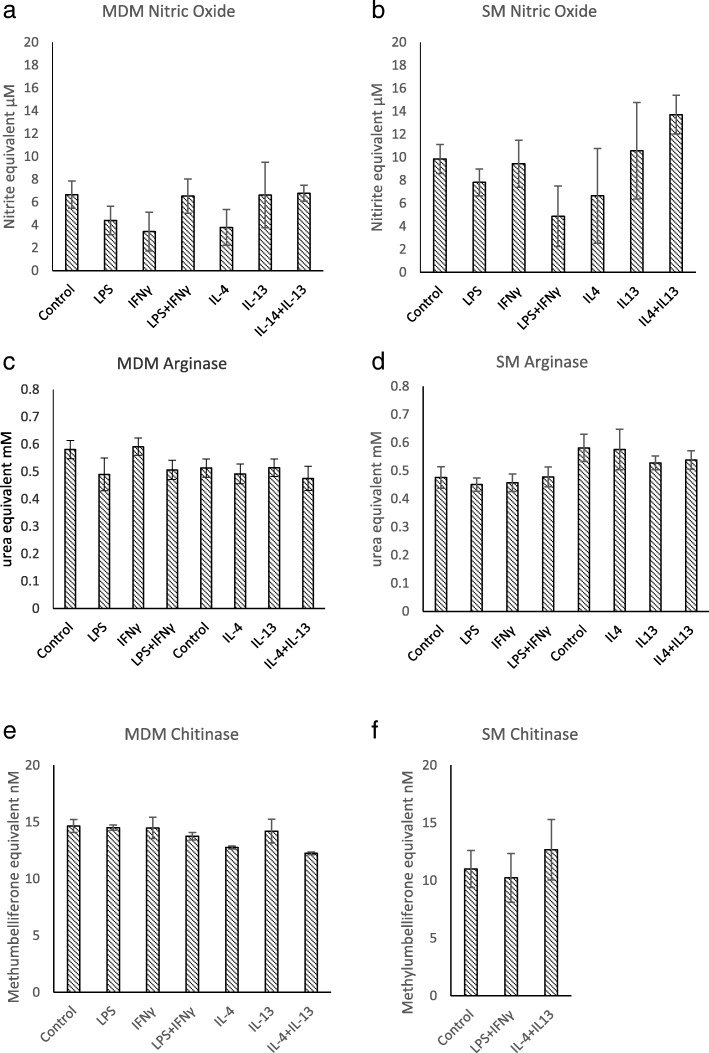
Levels of nitric oxide (1a & 1b) and chitinase (1e & 1f) in monocyte-derived and splenic macrophage cell supernatants and arginase (1c & 1d) in cell lysates following stimulation with LPS and/or cytokines (LPS 1000 ng/ml, IFNɣ 20 ng/ml, IL-4 20 ng/ml and IL-13 20 ng/ml) for 40 h. Levels of enzyme product were determined by measuring OD values and comparison with standard curves. Means and standard errors of data from triplicate cultures from representative individual animals are shown, monocyte-derived macrophages all being obtained from one animal and splenic macrophages all from another individual. Similar findings were recorded from all individuals tested (*n* = 5 MDM, *n* = 7 SM) for stimulation times of 12–72 h

LPS and IFNγ (M1 stimulants), either alone or in combination, did not significantly affect the detection of nitric oxide (a marker of M1 polarisation) or of arginase-1 and acidic chitinase by MDM from any of the individuals sampled. Representative findings from one individual are represented as means ± S.E. in Fig. 1a, c and e. Similarly IL-4 and IL-13 (M2 stimulants) alone or in combination did not affect production of nitric oxide, arginase-1 or acidic chitinase in MDM preparations (Fig. 1b, d and f). Similar results were found following stimulation of SM preparations (Fig. 1b, d and f). Data from triplicate wells were normally distributed and no significant differences were associated with any of the treatments (*p* > 0.1 for all comparisons).

#### Il-6

**Fig. 2:**
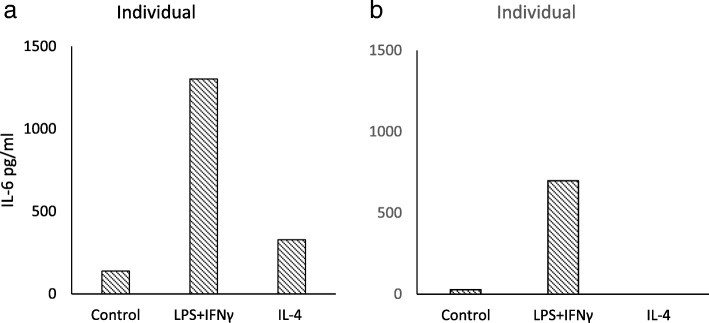
IL-6 concentration in supernatants from splenic macrophages stimulated with LPS (1000 ng/ml) and IFNγ (20 ng/ml) or IL-4 (20 ng/ml) for 20 h. OD values were determined from samples comprising combined supernatants from triplicate wells, run in duplicate. Results for two individual animals (**a** and **b**) are shown

LPS and IFNγ together were found to increase IL-6 production; IL-4 induced only a modest increase in IL-6. Findings from two individuals are shown in Fig. 2.

### Effect of stimulation of MDM with cytokines on transcription of enzymatic markers of M1 and M2 polarisation

**Fig. 3:**
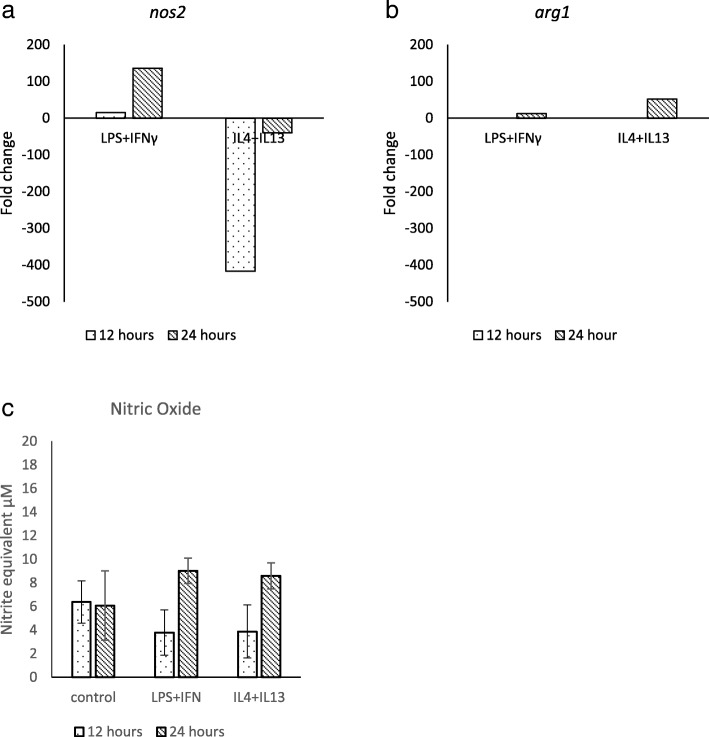
Fold changes in MDM production of *nos2* (**a**) and *arg1* (**b**) following stimulation with LPS (1000 ng/ml) & IFNγ (20 ng/ml) or IL-4 (20 ng/ml) & IL-13 (20 ng/ml) compared with changes in control cells, normalised with reference to transcription levels of GAPDH at 12 & 24 h. Fold changes in levels of nitric oxide in MDM cell supernatants from stimulated cells in comparison to unstimulated controls are shown in **c**. Supernatants and mRNA were harvested contemporaneously from the same cell populations and were performed in triplicate. Data from the same representative individual are shown in **a**, **b** and **c**

In order to investigate whether transcription patterns matched findings obtained using the Greiss reagent and arginase assays, MDM were stimulated with LPS and IFNγ or with IL-4 and IL-13 for 12–24 h. The change in transcription levels of *nos2* and *arg1* in stimulated and unstimulated controls were compared to the change in transcription level of the reference gene *gapdh*. Levels of *arg1* transcription were very low compared to other genes and were undetectable at 12 h. LPS and IFNγ stimulation increased transcription of *nos2* > 100-fold and *arg1* > 10-fold at 24 h (Fig. 3). IL-4 and IL-13 stimulation increased transcription of *arg1* approximately 50-fold at 24 h but decreased transcription of *nos2* > 400-fold. Inhibitory effects were greater at 12 h, whereas stimulatory effects were greater at 24 h (Fig. 3). Levels of NO in cell supernatants from stimulated MDM were not significantly different from those of control cells.

## Discussion

In the current study, it was not possible to detect increased production of the M1 marker NO, nor the M2 markers arginase and chitinase from MDM or SM harvested from healthy cattle and treated in vitro with pro- or anti-inflammatory stimulants. This is in contrast to the findings of others. Increased NO of the order of 20-60 μM nitrite equivalent has been found in similar systems following stimulation of in vitro MDM with LPS ± IFNɣ in mouse (reviewed [7, 13]), man [17, 18] [19] and cattle [20–24]. However, other results have been less clear-cut; Gibson et al. found that bovine MDM did not show a significant increase in NO activity in response to stimulation with LPS (although these cells were able to respond significantly to zymosan) [25]. Results relating to IFNγ also vary; in some studies IFNγ was found to stimulate a small increase in bovine MDM NO activity [26, 27] [28] whereas others have found no such increase [29]). In the current study, stimulation with LPS and IFNγ increased SM production of IL-6 suggesting that these cells did increase production of this pro-inflammatory cytokine in the face of TLR4 signalling and thus did demonstrate some features of typical M1 macrophages.

The activity of arginase in human MDM cell lysates has been detected by some authors [30] but not others [16]. Bovine MDM cell have been shown to produce both arginase [21, 31, 32] and chitinase activity [21] following simulation with IL-4. However, in the present study, stimulation of bovine MDM and SM with IL-4 and/or IL-13 did not increase arginase activity in cell lysates or chitinase activity in supernatants. This was surprising since the experimental methods used were similar to those of others [21, 23].

The contradictory results reported by different groups using similar methodologies suggests that, in addition to species differences, there are strain and/or individual differences in macrophage responses to simulation. This has been substantiated by the findings of others; inbred strains of mice vary greatly in their ability to increase iNOS activity [16, 33]. Stimulation of bovine MDM with LPS and IFNγ resulted in 2-fold increases in NO activity when harvested from animals which were resistant to *M. bovis* but not from susceptible animals [23]. Similarly, Brown Swiss cattle stimulated by bacterial infection produce more reactive nitrogen species than Holstein-Friesians which up-regulate more reactive oxygen species [25]. In contrast to this study, Flynn and Mulcahy (2008) found that macrophages obtained from castrated Friesian calves raised in indoor experimental conditions did exhibit increased NO activity in response to LPS [21]. These different findings may be related to genetic variation between breeds or to differences in environmental challenges; the animals utilised in this study were representative of those living in the farm setting, being mature Dexter and beef breeds with exposure to a variety of local microbes and parasites.

Individual variation in macrophage NO and arginase-1 expression may not be due to a lack of *nos2* and *arg1* gene transcription, but rather a lack of translation or increased inactivation of the protein products. In the current investigation, stimulation of MDM with LPS and IFNγ increased transcription of *nos2* more than 100-fold and IL-4 and IL-13 stimulation reduced *nos2* transcription by more 400-fold, whilst no significant difference was detected in NO activity in cell supernatants. It may be that the culture conditions and/or the cell type were lacking in one or more factors required for iNOS expression or NO activity [13]. Alternatively individual animals may show variation in levels of protein translation or catabolism. A possible mechanism involves the cofactor tetrahydrobiopterin (BH4) which is required for NO production by iNOS; it has been shown that when BH4 levels are limited, iNOS produces superoxide rather than NO [34]. Human macrophages, and possibly bovine, produce much less BH4 than murine [34].

In the current study, stimulation of MDM with LPS and IFNγ increased transcription of *arg1* greater than 10-fold and stimulation with IL-4 and IL-13 increased transcription greater than 50-fold. The ability of the anti-inflammatory cytokines IL-4 and IL-13 to increase transcription of *arg1* is well documented in mouse [35] and has been reported in bovine MDM [32]. Although LPS and IFNγ are associated with pro-inflammatory responses, they were found to up-regulate transcription of *arg1* which has been associated with anti-inflammatory responses. Interestingly, it has been found in mouse that it is the presence of the pro-inflammatory cytokine TNFα, rather than IFNγ, which is critical to the decreased transcription of *arg1* [36]. In rodents, although low dose LPS inhibits *arg1* transcription, higher doses have the opposite effect due to the activation of different pathways [37, 38]. LPS and IFN have been shown to induce *arg1* and it has been suggested that the activity of both arginase-1 and iNOS may damp down excessive inflammation [10, 39]. In the current study, once again there was a mismatch between detection of mRNA and enzymatic activity. However, levels of transcription of *arg1* were much lower than other genes investigated by quantitative RT-PCR, which suggests that levels of protein might be expected to be very low, even in stimulated cells.

## Conclusions

The results of this study suggest that treatment of bovine MDM with pro- and anti-inflammatory stimulants does not always result in detectable up-regulation of NO or arginase activity. Furthermore, assays which measure the activities of these enzymes do not always relate to quantitative RT-PCR measurement of transcription. In contrast to rodent macrophages, but in common with human cells, findings from bovine macrophages vary between studies; this may relate to subtle variation in transcriptional control or other post-transcriptional differences.

## Methods

### Cattle

Whole blood (Lithium heparin-anti-coagulated) was obtained from Seralab (UK) or from healthy animals at slaughter from a local abattoir and was used for purification of MDM. SM were extracted from spleens obtained from healthy cattle at slaughter. The cattle were of a variety of breeds (Seralab supplied blood from Dexters; blood and spleen samples collected at the abattoir were from several beef breeds) and included both males and females. Animals were over 18 months of age and had been raised in farm, rather than experimental, conditions.

### Cell culture

#### Monocyte-derived macrophages (MDM)

Mononuclear cells were separated by density gradient centrifugation [40]; whole blood was centrifuged at 300 g for 15 min, Buffy coats were diluted 1:1 in warmed Dulbecco’s phosphate-buffered saline (D-PBS; Sigma, UK) and over-layered onto Ficoll-Paque (Histopaque-1077; Sigma, UK), then centrifuged at 400 g for 30 min. Contaminating red cells were removed using erythrocyte lysis buffer (ELB) containing 0.15 M ammonium chloride [41]. Cells were then labelled with anti-human CD14 conjugated to magnetic beads (Miltenyi Biotec) according to the manufacturer’s instructions and passed over magnetic columns to isolate CD14^+^ cells [31, 42]. Following this process, numbers of dead cells were negligible (determined by Trypan blue). Cells were suspended in RPMI with glutamine (Sigma UK) supplemented with 10% FCS (Sigma), containing 200 U/ml penicillin (Sigma) and 200 mg/ml streptomycin (Sigma) at 5–20 x 10^5^cells/ml (1–4 x 10^5^cells/well) in 96-well plates (Costar) in triplicate for biochemical assays or in 24-well plates (Costar) for RNA extraction (3–4 × 10^6^ cells/well). Cells were incubated at 37 °C in 5% CO_2_, medium being replaced every 2 days, until cells achieved 80% confluence and they had matured into monocyte-derived macrophages (MDM), at 6–12 days culture.

#### Splenic CD14^+^ macrophages (SM)

Pieces of spleen approximately 2cm^3^ were collected into D-PBS with 2 mM EDTA, incised and incubated in 1 mg/ml collagenase D for 30–60 min at 37 °C, then progressively forced through 100 μm and 40 μm cell strainers into D-PBS-EDTA. Cell suspensions were over-layered onto Histopaque-1077, then treated with ELB and macrophages separated using CD14 beads. Numbers of dead cells, estimated using Trypan blue, were negligible. Flow cytometry following direct immunofluorescence staining with anti-bovine CD14-FITC (a gift from John Graham-Brown, University of Liverpool) showed that 84% of SM were CD14^+^. SM were placed in culture at 5–10 × 10^5^ cells/ml in 200 μl in 96-well plates in triplicate. Cells were cultured overnight prior to simulation.

Following incubation, both MDM and SM were found to be spindle-shaped or rounded with multiple processes. Labelling with mouse anti-bovine MHCII-FITC (Serotec) followed by flow cytometry showed that 93% MDM and 90% SM expressed MHCII.

### Cell stimulation

MDM or SM were stimulated by addition of 50–2000 ng/ml LPS from *E. coli* 0111:B4 (Insight Biotechnology Ltd., Middlesex) and/or 20-100 ng/ml bovine IFNγ (ThermoFisher Scientific, UK) or with 10-40 ng/ml recombinant bovine IL-4 (Kingfisher Biotech Inc., St Paul) and/or 10-20 ng/ml recombinant bovine IL-13 (Kingfisher Biotech Inc). Numbers of samples from individual animals are detailed in Table 1.

**Table 1:** Numbers of samples treated with M1 and M2 stimulants

Cell type	Stimulants	Number (n) of individual blood samples	Number (n) of Individual spleen samples
M1 Stimulants	LPS	5	7
	IFNγ	1	6
	LPS & IFNγ	3	3
M2 Stimulants	IL-4	4	6
	IL-13	1	2
	IL-4 & IL-13	2	3

Following stimulation for varying periods of time (12–72 h), supernatants (200 μl) were collected and stored at -20 °C for NO, chitinase and IL-6 assays. Cells in 96-well plates were lysed with 100ul 1% Triton X-100 (Sigma) in PBS for 20 min at room temperature and lysates stored at -20 °C prior to arginase assay. Cells in 24-well plates were used for RNA extraction.

### Biochemical assays

#### Nitric oxide assay

Production of nitric oxide in cell supernatants was measured using the Greiss reagent system (Promega) according to the manufacturer’s instructions. Samples from each of the triplicate wells were measured in duplicate (standards were run in triplicate). Mean OD values were used to calculate the concentration of nitrite in supernatants using the standard curve. Results were expressed as μM nitrite equivalent. The limit of detection stated by the manufacturer was 2.5 μM nitrite.

#### Arginase assay

The arginase assay was performed as previously described [21, 43, 44]. Reagents were obtained from Sigma-Aldrich (UK). Cell lysate (50 μl) and 10 mM MnCl_2_/50 mM Tris-HCl (pH 7) (50 μl) were incubated at 55 °C for 10 min prior to the addition of 50 μl of 0.5 M L-arginine substrate and incubation at 37 °C for 1 h. Beef liver homogenate was used as a positive control and standards of the urea product (0–2 mM) were prepared. The reaction was stopped by addition of 400 μl acid-stop solution, comprising H_2_SO_4_ (96%), H_3_PO_4_ (85%), and H_2_O in a ratio of 1:3:7 and colour was developed by addition of 25 μl 9% isonitrosopriopherone. The mixture was heated to 100 °C for 45 min and 200 μl volumes transferred to a 96-well plate and OD540nm measured. Standards were prepared in triplicate and lysates from each of the triplicate sample wells were measured in duplicate. Mean OD values were used to determine urea concentrations using the standard curve. Results were expressed as mM urea equivalent.

#### Chitinase assay

Acidic chitinase concentration in supernatants was determined as previously described [44, 45]. Reagents were obtained from Sigma-Aldrich (UK). Aliquots (10 μl) of cell supernatant were incubated at 37C for 60 min with 40 μl McIlvaine buffer (0.1 M citric acid and 0.2 M sodium phosphate) containing 0. 25 mM of 4-methylumbelliferyl β-D-N,N′-diacetylchitobiose (4MU-chitobiose) substrate. Standards of 0-100 nM 4-methylumbelliferone (4MU) were prepared. 200 μl of 0.25 M glycine/NaOH was added to standards and samples to stop the reaction. Fluorescence (excitation 365 nm; emissions 460 nm) was determined. Standards were prepared in triplicate and supernatants from each of the triplicate sample wells were measured in duplicate. Mean OD values were used to determine methylumbelliferone concentrations using the standard curve and results expressed as nM methylumbelliferone equivalent.

### Interleukin-6 assay

Levels of the pro-inflammatory cytokine IL-6 in pooled supernatants from triplicate wells containing SM (*n* = 2) were determined using a Bovine IL-6 reagent kit (Thermo Scientific) according to manufacturers’ instructions. Samples were run in duplicate.

### Quantitative RT-PCR

mRNA was extracted from cultured MDM using RNAqueous Micro-kit (Ambion) according to the manufacturer’s instructions and treated with DNase (Ambion) and DNase inhibitor (Ambion). Single strand cDNA was synthesised using the GoScript Reverse Transcription Kit (Promega) according to the manufacturer’s recommendations. iTaq Universal SYBR® Green Supermix (Bio-Rad) and forward and reverse primers (Sigma) (final concentration of each 0.5pM) were prepared in triplicate in 96 well plates prior to addition of cDNA diluted 1 in 10. Q-PCR performed using LightCycler 480 (Roche). Primer sequences and efficiencies are detailed in Table 2.

**Table 2:** Primer details [47]

Gene	Forward Sequence	Reverse Sequence	No. bp	Efficiency
*nos2*	TTGAGATCAACGTCGCTGTG	CATGATGGTCACGTTCTGCT	56	98
*arg1*	ATGTGGACCCTGGGGAAC	ATGTTTCTTCCATCACCTTGC	105	101
*gapdh*	CACCATCTTCCAGGAGCGAG	CCAGCATCACCCCACTTGAT	51	100

The measured change in transcription levels of *nos2* and *arginase-1* following stimulation was compared with that of the reference gene, glyceraldehyde 3-phosphate dehydrogenase (*gapdh*) and fold changes were calculated using the delta-delta C_t_ method where the ratio R is determined by the equation below [46].

$$ \mathrm{R}={2}^{\hbox{-} \left\{\Delta \mathrm{Cp}\ \mathrm{sample}-\Delta \mathrm{Cp}\ \mathrm{reference}\right]} $$

### Statistical analysis

Measured values of nitrite, urea and methylumbelliferone from triplicate cultures were assessed for normality using the Shapiro-Wilk test and data were analysed using ANOVA and Tukey post hoc test to determine if any of the treatments was associated with a significant change (*p* < 0.02).

## Abbreviations

D-PBS: Dulbecco’s phosphate-buffered saline
EDTA: Ethylenediaminetetraacetic acid
H_2_SO_4_ sulphuric acid: H_2_PO_4_ phosphoric acid
IFNɣ: Interferon-gamma
IL: Interleukin
iNOS: inducible nitric oxide synthase
LPS: Lipopolysaccharide
MDM: Monocyte-derived macrophages
MHC: Major histocompatibility complex
NaOH: sodium hydroxide
NO: Nitric oxide
OD: Optical density
RT-PCR: Real time polymerase chain reaction
SM: Splenic macrophages
Tris-HCl: Tris(hydroxymethyl)aminomethane-hydrochloric acid
